# Right‑DLPFC rTMS Transiently Modulates Risky Decision‐Making and EEG Oscillatory Activity

**DOI:** 10.1002/brb3.71584

**Published:** 2026-07-15

**Authors:** Reza Kazemi, Reza Rostami, Maryam Majidinezhad, Hamed Hosseini Zarrabi, Sanaz Khomami, Mehdi Rezaei, Ahmad Zandbagleh

**Affiliations:** ^1^ Faculty of Entrepreneurship University of Tehran Tehran Iran; ^2^ Department of Psychology University of Tehran Tehran Iran; ^3^ Department of Clinical Psychology School of Behavioral Sciences and Mental Health (Tehran Institute of Psychiatry) Iran University of Medical Sciences Tehran Iran; ^4^ Department of Psychology, West Tehran Branch Islamic Azad University Tehran Iran; ^5^ Department of Psychology, Faculty of Educational Sciences and Psychology University of Birjand Birjand Iran; ^6^ Institute of Psychology Jagiellonian University Kraków Poland; ^7^ School of Electrical Engineering Iran University of Science and Technology Tehran Iran

**Keywords:** alpha asymmetry, EEG, Iowa Gambling Task, right dorsolateral prefrontal cortex, risky decision‐making, rTMS

## Abstract

**Background:**

Previous evidence suggests that inhibition of the right dorsolateral prefrontal cortex (DLPFC) increases risky decision‐making, highlighting its role in cognitive control and risk aversion. However, it remains unclear whether excitatory stimulation of this region can reduce risky decision‐making. Combining repetitive transcranial magnetic stimulation (rTMS) with Electroencephalography (EEG) provides an opportunity to examine neurophysiological correlates of stimulation‐related changes in decision‐making.

**Objective:**

This study aimed to examine whether high‐frequency (20 Hz) rTMS applied over the right DLPFC influences decision‐making performance and associated EEG measures in healthy adults.

**Methods:**

A within‐subject, crossover design was employed. EEG recordings were obtained before and after stimulation, while the Iowa Gambling Task (IGT) was administered at three stages: baseline, during stimulation, and post‐stimulation. EEG was recorded during both eyes‐closed and task‐performance conditions.

**Results:**

During active rTMS, participants showed reduced IGT performance, reflected by a decrease in advantageous card selections. Performance returned to baseline following stimulation, whereas no comparable changes were observed during sham stimulation. EEG analyses revealed a significant post‐stimulation increase in β‐band power following active stimulation. No stimulation‐related effects were observed for frontal theta activity, theta/beta ratio (TBR), or frontal alpha asymmetry (FAA), although a reduction in frontal TBR was observed during the sham–task condition.

**Conclusion:**

The findings provide preliminary evidence that high‐frequency rTMS over the right DLPFC can transiently influence adaptive decision‐making performance and is associated with post‐stimulation modulation of β‐band activity. The dissociation between behavioral effects observed during stimulation and EEG changes measured after stimulation suggests that neurophysiological modulation may persist beyond the period of behavioral interference. Given the small sample size and exploratory nature of the analyses, these findings should be considered preliminary and require replication in larger samples.

## Graphical abstract

High‐frequency (20 Hz) rTMS over the right dorsolateral prefrontal cortex transiently altered decision‐making in healthy adults. During stimulation, selection of advantageous options decreased without an increase in risky choices, indicating changes in adaptive strategy rather than impulsivity. EEG results showed increased post‐stimulation beta power, supporting the role of the right DLPFC in adaptive decision‐making performance.

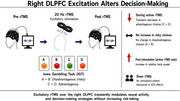

## 1 Introduction

Successfully navigating complex environments requires the ability to make advantageous decisions, a process that involves weighing immediate rewards against long‑term consequences (Ernst and Paulus 2005). This complex evaluation is not merely a behavioral tendency but reflects a core function of the brain's executive control systems, constituting a cornerstone of adaptive cognition (Miller and Cohen 2001). Importantly, the clinical stakes are high, as impairments in decision‑making are not peripheral symptoms but represent a core transdiagnostic feature. Such impairments have been well documented across a broad range of psychiatric and behavioral disorders, including substance use disorders (Chen et al. 2020), gambling disorder (Kovács et al. 2017), schizophrenia (Fond et al. 2013), bipolar disorder (Adida et al. 2011), and obsessive–compulsive disorder (Nisticò et al. 2021). Understanding the neural mechanisms that underpin these choices is therefore critical for both basic cognitive neuroscience and translational clinical applications.

While neuroimaging can identify brain regions associated with a given behavior, it cannot by itself establish causal relationships. Noninvasive brain stimulation techniques provide an important approach for addressing this limitation, allowing researchers to directly manipulate neural activity and observe the resulting behavioral consequences. Indeed, a series of foundational studies used low‑frequency (1 Hz) repetitive transcranial magnetic stimulation (rTMS), which is commonly employed to transiently reduce cortical excitability. These experiments provided causal evidence suggesting a hemispheric asymmetry in the dorsolateral prefrontal cortex (DLPFC) in decision‑making. Across studies, disrupting the right DLPFC led to increased risk‑taking behavior during gambling paradigms (Knoch et al. 2006) and other strategic decision tasks (Tulviste and Bachmann 2019; Van't Wout et al. 2005). Together, these findings suggest that the right DLPFC contributes to the top‑down regulation of impulsive or immediately rewarding choices (Knoch et al. 2006; Tulviste and Bachmann 2019; Van't Wout et al. 2005).

The functional profile of the left DLPFC, however, appears to show an opposing pattern. Studies using excitatory stimulation have shown that increasing left DLPFC excitability enhances reward responsiveness (Ahn et al. 2013) and promotes more patient, value‑based choices (Xiong et al. 2019). Conversely, low‑frequency rTMS applied to the left DLPFC increases the preference for smaller, immediate rewards, implying reduced capacity for delayed‑reward evaluation (Figner et al. 2010). In light of these findings, these findings raise a critical and unresolved question: if transient disruption of the right DLPFC increases risk‑taking, what are the behavioral consequences of enhancing its excitability using high‑frequency rTMS? Specifically, might increasing activity in this region strengthen top‑down inhibitory control mechanisms, thereby biasing individuals toward more cautious and risk‑averse decision‑making?

To address this question, it is essential to consider the neural oscillatory mechanisms implicated in risky decision‑making. A substantial body of electrophysiological research has identified several frequency‑specific indices that predict individual differences in risk propensity. Foundational work on frontal alpha asymmetry (FAA) established it as a key marker of approach–avoidance motivation (Davidson 1992; Tomarken et al. 1992), grounded in the widely supported assumption that alpha power inversely reflects cortical activation (Coan and Allen 2004; Harmon‐Jones and Allen 1997). Complementing this, greater resting‑state asymmetry in slow‑wave bands (theta and delta) has been shown to predict increased risk‑taking tendencies (Gianotti et al. 2009; Studer et al. 2013). Furthermore, the theta/beta ratio (TBR) has emerged as a robust index of motivational balance and decision bias, originally linked to reward–punishment sensitivity and later associated with disadvantageous choices in risky contexts (Schutte et al. 2017; D. J. Schutter and Van Honk 2005). Interventional studies aimed at probing the causal role of these oscillations have yielded divergent outcomes, particularly within the beta range: while 20‑Hz tACS can enhance cognitive flexibility during adaptive learning (Wischnewski et al. 2020), the same frequency has been found to increase risk‑taking when applied over the left PFC (Yaple et al. 2017). These inconsistencies—suggesting that beta‑band modulation may simultaneously facilitate cognitive control yet promote risk—underscore the complexity of predicting its behavioral effects in paradigms such as the IGT. Crucially, 20‑Hz stimulation has also been shown to modulate relationships between frequency bands, including the TBR (Wischnewski et al. 2020), highlighting its broader network‑level impact. Rhythmic TMS further strengthens this rationale: accumulating evidence demonstrates that rhythmic stimulation can entrain endogenous oscillations in a frequency‑specific manner, realigning intrinsic rhythms toward the stimulation frequency (Thut et al. 2011). Recent reviews indicate that rTMS can entrain beta‑band activity in both motor and prefrontal regions, with meaningful behavioral consequences (Wang et al. 2024). Notably, β‑rhTMS has been shown to entrain sensorimotor beta oscillations and modulate motor vigor (Uehara et al. 2023), providing causal support for stimulation within the beta range effectively interacting with intrinsic beta dynamics. Given that beta oscillations are increasingly conceptualized as reflecting active inhibitory processes that stabilize ongoing cognitive states (Engel and Fries 2010), and considering the role of the right DLPFC in exerting such control, beta emerges as a theoretically precise primary target for neuromodulation. Additionally, due to its foundational role in approach–avoidance motivation, FAA serves as a critical secondary index for capturing hemispheric and motivational effects of unilateral stimulation (Davidson 1998).

The present study was designed to directly address this open question. Using a rigorous sham‑controlled within‑subjects design, we investigated how 20‑Hz rTMS applied to right DLPFC influences performance on the Iowa Gambling Task (IGT). We specifically hypothesized that modulating the cognitive‑control network—indexed by beta‑band oscillations—in the right DLPFC would alter the participant's motivational state. We predicted that enhanced engagement of the right DLPFC would bias participants toward more cautious decision‑making on the IGT and would be accompanied by a shift in FAA, reflecting an altered motivational balance.

## 2 Materials and Methods

### 2.1 Participants

Following a recruitment announcement distributed on social media platforms in July 2024, 34 individuals expressed interest in participating in the study. All respondents completed an online version of the Symptom Checklist‐90 (SCL‐90) (Derogatis et al. 1973) as a preliminary screening measure. Based on SCL‑90 scores and a subsequent clinical interview conducted by a trained researcher, 12 candidates met the eligibility criteria and were invited to participate in the experimental sessions. After confirmation of inclusion and exclusion criteria, the sample size was calculated using G*Power (Version 3.1.9.2, Kiel, Germany) software as follows: test family = *F*‐tests; Statistical test = MANOVA: repeated measures, within factors; *α* error probability = 0.05; power (1‐β err prob) = 0.80; Effect size *f* = 0.5, number of groups = 1, number of measurements = 8, correlation among repeated measures = 0.5. Accordingly, 12 participants (six males, six females) were selected as the sample size for the present study.

Inclusion criteria required participants to be between 18 and 40 years of age, possess university‐level education, and be right‐handed, as verified by the Edinburgh Handedness Inventory (Oldfield 1971). Additional criteria were an SCL‐90 Global Severity Index score below the clinical cutoff of 1.3, absence of prior exposure to noninvasive brain stimulation techniques (e.g., rTMS, tDCS), and the provision of written informed consent. Exclusion criteria included any current or past psychiatric or neurological disorders (e.g., epilepsy, seizure history, traumatic brain injury), presence of metallic implants in the head, use of a cardiac pacemaker, or unwillingness to continue participation at any point. These criteria were determined in accordance with international rTMS safety guidelines (Rossi et al. 2009). All participants were screened for TMS safety using a standardized checklist and were informed about the study procedures, potential risks, and their right to withdraw at any time. The study protocol was approved by the Ethics Committee of the Faculty of Psychology and Education at the University of Tehran under approval code IR.UT.PSYEDU.REC.1403.058.

### 2.2 Procedure

The experiment was conducted between August and October 2024 at the Atieh Clinical Neuroscience Center (Tehran, Iran). The study employed a randomized, placebo‑controlled, double‑blind, within‑subject crossover design. Each participant completed two sessions—active 20‐Hz rTMS and sham stimulation—with the order of conditions randomly assigned using a computer‐generated sequence. Both participants and the experimenter responsible for EEG acquisition and behavioral testing remained blinded to the stimulation condition. At the beginning of each session, participants were seated in an EEG laboratory. A pre‑stimulation baseline EEG was recorded during (i) an eyes‑closed resting‑state period and (ii) task performance during the IGT. Immediately thereafter, the assigned stimulation protocol (active or sham) was administered. During the stimulation period, participants performed the IGT to assess potential online modulation of risk‐related decision‐making. Upon completion of the stimulation phase, a post‐stimulation EEG was recorded following the same sequence as baseline—eyes‐closed resting‐state EEG and task‐related EEG during a subsequent IGT session—to quantify offline neurophysiological aftereffects of rTMS. In the sham rTMS condition, the same stimulation parameters and scalp positioning were used; however, the coil was angled at 90° away from the scalp, with one edge resting on the head. This approach preserves, to a large extent, the auditory clicking and somatosensory scalp sensations associated with active stimulation while minimizing effective cortical current induction. This method has been widely used in prior rTMS studies on decision‐making (e.g., Knoch et al. 2006) and is considered a valid procedure for maintaining participant blinding. Importantly, both active and sham conditions followed identical timing and procedural structures, thereby controlling for nonspecific sensory effects during task performance.

### 2.3 Experimental Task

Decision‐making under uncertainty was assessed using a computerized version of the IGT, modeled after the original paradigm (Bechara et al. 1994). On each trial, participants selected one card from four simultaneously presented decks (A, B, C, and D) with the overarching goal of maximizing their virtual monetary outcomes across 100 trials. The payoff schedule was structured to induce a trade‐off between immediate reward and long‐term profitability. Decks A and B were classified as disadvantageous, yielding large immediate gains (€100) but paired with occasional substantial losses that produced a net negative outcome over repeated selections. In contrast, decks C and D were advantageous, offering smaller immediate gains (€50) accompanied by modest penalties, resulting in a net positive outcome across the task. The primary behavioral index was the IGT net score, computed as the number of selections from advantageous decks minus disadvantageous decks: net Score = (C + D) − (A + B). To examine learning dynamics, performance was further segmented into five blocks of 20 trials, enabling assessment of changes in choice patterns across task progression. The task was administered in identical form during baseline, online stimulation, and post‐stimulation phases, allowing direct comparison of decision‐making performance across experimental conditions.

### 2.4 rTMS

rTMS was delivered using a Magstim Rapid^2^ stimulator (Magstim Co., Whitland, UK) equipped with a standard figure‐of‐eight coil. Stimulation was applied over the right DLPFC, operationally defined as the F4 position of the international 10–20 EEG system. Each participant's resting motor threshold (RMT) was determined over the left primary motor cortex. The motor hotspot for the abductor pollicis brevis (APB) muscle was identified as the scalp position that produced a visible thumb contraction in at least five out of 10 consecutive pulses. RMT was defined as the lowest stimulation intensity achieving this criterion. Participants received a 20‐Hz high‐frequency rTMS protocol. Stimulation consisted of 70 trains, each lasting 2 s and containing 40 pulses, delivered with an inter‐train interval of 17 s. This amounted to a total of 2800 pulses, with the entire stimulation session lasting approximately 22 min. All parameters were selected in accordance with contemporary safety guidelines for high‐frequency TMS (Rossi et al. 2009). The sham condition matched the active stimulation session in coil placement, timing, and train structure. However, the coil was positioned at a 90° angle relative to the scalp, with one rim resting lightly on the head. This configuration reproduces the auditory and somatosensory sensations associated with active stimulation while preventing effective cortical induction. This sham approach has been widely adopted in prior rTMS studies of decision‐making (e.g., Knoch et al. 2006) and is recognized as a valid method for maintaining participant blinding.

### 2.5 EEG Recording and Analysis

#### 2.5.1 EEG Acquisition

Continuous electroencephalographic (EEG) activity was recorded using a 21‐channel Mitsar‐201 system (Mitsar Co., St. Petersburg, Russia) operated with WinEEG software. EEG signals were acquired with a standard elastic cap containing 21 Ag/AgCl electrodes positioned according to the international 10–20 system. Data were recorded with a linked‑ears (A1–A2) reference and retained with the same reference during offline processing. Data were sampled continuously at 500 Hz, and electrode impedances were maintained below 5 kΩ throughout the recording by applying conductive gel and adjusting electrode contacts as necessary. In both the active rTMS and sham sessions, two EEG recordings were collected: (i) pre‐stimulation and (ii) post‐stimulation. Each recording block began with a 5‑min eyes‑closed resting‑state period, immediately followed by task‑related EEG during IGT performance.

#### 2.5.2 EEG Preprocessing and EEG Analysis

Offline EEG processing was performed using the WinEEG software suite, following established procedures for spectral and artifact‐corrected EEG analysis. Raw continuous data were first filtered using a 0.5‐Hz high‐pass filter and a 45‐Hz low‐pass filter to avoid overly sharp transitions near the cutoff frequency. A notch filter centered around 50 Hz was additionally applied to attenuate residual power line noise in this frequency range that may persist after low‐pass filtering. Preprocessing proceeded in two stages. First, segments containing gross, non‐stereotyped artifacts (e.g., movement‐related bursts, electrode pops) were identified through visual inspection and removed. Second, independent component analysis (ICA) was applied to the remaining dataset to isolate and remove components associated with stereotyped physiological artifacts, including eye blinks, saccades, and muscle activity. Components were rejected based on their scalp topographies, time courses, and spectral characteristics. The cleaned continuous recordings were then segmented into artifact‐free 4‐s epochs. For quantitative EEG (qEEG) analysis, each epoch underwent a fast Fourier transform (FFT) to compute the power spectral density (PSD) for each electrode. Spectral estimates were subsequently averaged across epochs within each condition (pre‐stimulation and post‐stimulation, for both active and sham sessions). This preprocessing pipeline ensured high‐quality, artifact‐reduced spectral data suitable for examining frequency‐specific neural dynamics, including beta‐band power changes associated with rTMS administration.

#### 2.5.3 EEG Analysis

To address the inherent nonstationarity of EEG signals, continuous artifact‐free data were segmented into nonoverlapping 4‐s epochs prior to spectral estimation. PSD was computed for each epoch using Welch's method, which provides a robust estimate of frequency‐domain power while reducing variance through segment averaging. Absolute spectral power was calculated for canonical frequency bands defined according to established boundaries: delta (1–4 Hz), theta (4–8 Hz), alpha (8–13 Hz), beta (13–30 Hz), and gamma (30–45 Hz). For each band, the relevant PSD frequency bins were integrated to derive the total absolute power. To facilitate comparison across participants and sessions, relative power values were obtained by normalizing absolute band power to the total broadband power across 1–45 Hz. This normalization yielded unitless measures representing the proportion of total spectral power allocated to each band. For each participant, relative power values were averaged across all epochs and computed separately for each electrode. Additionally, FAA was calculated using the standard log‐transformed ratio method, defined as follows: FAA = ln (α_F3) − ln (α_F4).

Where α_F3 and α_F4 denote the relative alpha‐band power at left (F3) and right (F4) frontal electrodes, respectively. This index provides a unidimensional measure of interhemispheric asymmetry, with values near zero indicating symmetrical activity, positive values reflecting greater right‐hemisphere alpha power (α_F4 > α_F3), and negative values indicating relatively stronger left‐hemisphere alpha power (α_F3 > α_F4). Given the inverse relationship between alpha power and cortical activation, positive FAA values are typically interpreted as relatively greater left frontal cortical activity, whereas negative values reflect relatively greater right frontal activity.

### 2.6 Statistical Analysis

All statistical analyses were performed using SPSS Version 27 (IBM SPSS Statistics; Chicago, IL, USA). The significance threshold was set at *α* = 0.05 (two‐tailed). The normality of all dependent variables was assessed using the Kolmogorov–Smirnov test. When violations of normality were detected, data were corrected using the Box–Cox transformation. Mauchly's test of sphericity was applied to repeated‐measures factors, and the Greenhouse–Geisser correction was used when the sphericity assumption was violated.

Behavioral data were analyzed using a three 2 × 3 repeated measures MANOVA, with condition (active rTMS, sham) and time (pre‐stimulation, during stimulation, post‐stimulation) served as within‐subject factors. Dependent measures included the following: selections from disadvantageous decks (A, B), selections from advantageous decks (C, D), and block‐by‐block IGT net scores across the five 20‐trial blocks. The primary behavioral outcome was the standard IGT net score (C + D) − (A + B). Significant main effects or interactions were followed by Bonferroni‐corrected pairwise comparisons. EEG outcomes were analyzed using four 2 × 2 × 2 repeated measures MANOVAs, with stimulation condition (active, sham), recording condition (rest, task), and time (pre‐stimulation, post‐stimulation) as within‐subject factors. When the multivariate *F* was significant, univariate *F* and then dependent samples *t*‐test with Bonferroni correction were conducted. Dependent variables included: EEG spectral power (absolute and relative) across frequency bands, FAA, and TBR.

To address the limited sample size (*N* = 12) and the high dimensionality of the EEG dataset (19 channels × multiple frequency bands), we reduced data dimensionality by averaging electrode activity within five predefined regions of interest (ROIs). The ROIs were as follows: frontal (Fp1, Fp2, F7, F3, Fz, F4, F8), central (C3, Cz, C4), temporal (T3, T4, T5, T6), parietal (P3, Pz, P4), and occipital (O1, O2). This regional averaging approach is commonly used in EEG research to improve statistical power and reduce the multiple‐comparison burden in studies with small sample sizes and multichannel recordings (Bowman et al. 2020).

## 3 Results

### 3.1 Behavioral Results

#### 3.1.1 Order/Sequence Effect

To examine sequence effects on behavioral variables and EEG data, respectively, the following mixed MANOVAs were conducted: a 2 × 2 × 3 × 2 mixed MANOVA and a 2 × 2 × 2 mixed MANOVA. Stimulation order (active‐first vs. sham‐first) served as a between‐subjects factor. Across all behavioral and neurophysiological dependent variables, the order of stimulation (active‐first vs. sham‐first) showed no significant main effects and did not enter into any significant interactions (all *p* > 0.10). Given the absence of order‐related influences on task performance or EEG measures, order was not included as a factor in subsequent analyses to improve model parsimony and statistical power. Table 1 presents the descriptive statistics of participants' scores on the behavioral task (IGT)

**TABLE 1:** Mean (± SD) selections from advantageous and disadvantageous decks across five IGT blocks in active and sham stimulation conditions.

	Active (Pre) (mean ± SD)	Sham (Pre) (mean ± SD)	Active (TMS) (mean ± SD)	Sham (TMS) (mean ± SD)	Active (Post) (mean ± SD)	Sham (Post) (mean ± SD)
A.B choices						
A.B choice. Block 1	9.16 ± 5.84	10.58 ± 3.42	10.66 ± 5.24	12.08 ± 4.54	8.25 ± 4.71	8.75 ± 3.62
A.B choice. Block 2	7.75 ± 4.95	9.41 ± 4.96	7.16 ± 4.78	7.58 ± 4.44	7.08 ± 5.23	7.66 ± 5.28
A.B choice. Block 3	6.50 ± 4.96	7.91 ± 5.03	8.58 ± 5.41	7.66 ± 5.85	6.08 ± 5.48	6.91 ± 3.87
A.B choice. Block 4	6.25 ± 4.24	7.41 ± 5.74	6.16 ± 5.47	6.25 ± 5.86	5.25 ± 4.67	7.25 ± 5.91
A.B choice. Block 5	5.00 ± 4.80	7.91 ± 5.63	6.00 ± 5.49	6.83 ± 5.09	6.75 ± 4.84	6.00 ± 5.44
C.D choices						
C.D choice. Block 1	10.83 ± 5.84	9.41 ± 3.42	9.33 ± 5.24	7.91 ± 4.54	11.75 ± 4.71	11.25 ± 3.62
C.D choice. Block 2	12.25 ± 4.95	10.58 ± 4.96	12.83 ± 4.78	12.41 ± 4.44	12.91 ± 5.23	12.33 ± 5.28
C.D choice. Block 3	13.50 ± 4.96	12.08 ± 5.03	10.25 ± 5.42	12.33 ± 5.85	13.91 ± 5.48	13.08 ± 3.87
C.D choice. Block 4	13.66 ± 4.35	12.58 ± 5.74	10.66 ± 4.69	13.91 ± 5.86	14.75 ± 4.67	13.58 ± 5.56
C.D choice. Block 5	15.00 ± 4.80	12.08 ± 5.63	9.83 ± 3.53	13.16 ± 5.09	13.25 ± 4.84	14.00 ± 5.44
Net scores						
Net score. Block 1	1.66 ± 11.68	−1.16 ± 6.84	−1.33 ± 10.49	−4.16 ± 9.08	3.50 ± 9.42	2.50 ± 7.24
Net score. Block 2	4.50 ± 9.91	1.16 ± 9.92	5.66 ± 9.56	4.83 ± 8.81	5.83 ± 10.46	4.66 ± 10.56
Net score. Block 3	7.00 ± 9.92	4.16 ± 10.07	2.83 ± 10.83	4.66 ± 11.70	7.83 ± 10.96	6.16 ± 7.74
Net score. Block 4	7.41 ± 8.59	5.16 ± 11.48	4.50 ± 8.78	7.50 ± 11.72	9.50 ± 9.34	6.33 ± 11.11
Net score. Block 5	10.00 ± 9.61	4.16 ± 11.26	3.83 ± 8.40	6.33 ± 10.19	6.50 ± 9.69	8.00 ± 10.88

*Note*: According to the Bonferroni correction, the significance level was set at *p* ≤ 0.010.

#### 3.1.2 Disadvantageous Deck Selections (A and B Decks)

A 2 × 3 repeated measure MANOVA was conducted to evaluate whether active right‐DLPFC rTMS influenced participants’ tendency to select cards from the disadvantageous decks (A, B) across the IGT. The repeated measure MANOVA showed that for the linear combination of five dependent variables (Block 1, 2, 3, 4, 5), there were no significant effects of time (Wilks' Lambda = 0.38, *F*_(2, 10)_ = 2.60, *p* = 0.122, *η* ^2^ = 0.39), Stimulation Condition (Wilks' Lambda = 0.63, *F*_(5, 7)_ = 0.81, *p* = 0.570, *η* ^2^ = 0.36), and the time × condition interaction (Wilks' Lambda = 0.67, *F*_(2, 10)_ = 1.11, *p* = 0.366, *η* ^2^ = 0.18). Subsequently, the univariate *F* showed that the time × condition interactions were not significant for any of the dependent variables (Table 2).

**TABLE 2:** The results of the repeated measure MANOVA.

Variables		Time				Condition				Time × condition		
	*F*	Df1, Df2	*p*	*η* ^2^	*F*	Df1, Df2	*p*	*η* ^2^	*F*	Df1, Df2	*p*	η^2^
A.B choices												
A.B choice. Block 1	7.29	2, 22	**0.004**	0.40	1.85	1, 11	0.204	0.14	0.16	2, 22	0.848	0.01
A.B choice. Block 2	2.48	2, 22	0.104	0.18	2.0	1, 11	0.177	0.15	0.33	2, 22	0.727	0.03
A.B choice. Block 3	3.01	2, 22	0.072	0.21	0.78	1, 11	0.405	0.06	1.18	2, 22	0.319	0.10
A.B choice. Block 4	0.38	2, 22	0.692	0.03	1.67	1, 11	0.222	0.13	0.63	2, 22	0.538	0.05
A.B choice. Block 5	0.02	2, 22	0.993	0.0	2.69	1, 11	0.129	0.19	2.47	2, 22	0.117	0.18
C.D choices												
C.D choice. Block 1	7.18	2, 22	**0.004**	0.39	1.85	1, 11	0.204	0.14	0.45	2, 22	0.643	0.04
C.D choice. Block 2	2.47	2, 22	0.104	0.18	2.0	1, 11	0.177	0.15	0.32	2, 22	0.727	0.03
C.D choice. Block 3	0.42	2, 22	0.659	0.04	0.79	1, 11	0.408	0.06	0.81	2, 22	0.457	0.07
C.D choice. Block 4	2.81	2, 22	0.077	0.21	9.65	1, 11	**0.010**	0.46	8.18	2, 22	**0.002**	0.42
C.D choice. Block 5	5.92	2, 22	**0.009**	0.35	10.10	1, 11	**0.009**	0.49	13.87	2, 22	**0.0001**	0.56
**Net Scores**										2, 22		
Net score. Block 1	6.16	2, 22	**0.008**	0.37	1.84	1, 11	0.204	0.14	0.17	2, 22	0.848	0.01
Net score. Block 2	2.51	2, 22	0.104	0.18	2.01	1, 11	0.177	0.15	0.32	2, 22	0.727	0.03
Net score. Block 3	0.42	2, 22	0.659	0.04	0.80	1, 11	0.408	0.06	1.29	2, 22	0.318	0.09
Net score. Block 4	0.16	2, 22	0.848	0.02	0.21	1, 11	0.666	0.02	2.57	2, 22	0.128	0.17
Net score. Block 5	0.49	2, 22	0.624	0.04	0.14	1, 11	0.708	0.01	4.57	2, 22	0.022	0.29

*Note*: Bold values indicate statistically significant results after Bonferroni correction (*p* ≤ 0.010).

#### 3.1.3 Advantageous Deck Selections (C and D Decks)

A 2 × 3 repeated measure MANOVA was conducted to examine whether active high‐frequency right‐DLPFC rTMS modulated participants’ selections from the advantageous decks (C, D) across the five IGT blocks. Overall, the results of the repeated measure MANOVA indicated that the main effect of time (Wilks' Lambda = 0.15, *F*_(2, 10)_ = 8.02, *p* = 0.008, *η* ^2^ = 0.62) and the time × stimulation condition interaction (Wilks' Lambda = 0.16, *F*_(2, 10)_ = 7.32, *p* = 0.010, *η* ^2^ = 0.59) were significant for the linear combination of the five dependent variables (Blocks 1, 2, 3, 4, 5). However, the main effect of Condition was not significant (Wilks' Lambda = 0.34, *F*_(7, 5)_ = 2.63, *p* = 0.125, *η* ^2^ = 0.65). Given the significant interaction effect, univariate *F*‐tests were conducted to follow up on significant differences across the two conditions (sham and active rTMS) and three time points (pretest, rTMS phase, and posttest). In this stage, given the multiplicity of dependent variables (five variables), the Bonferroni correction ($\frac{\alpha }{5}=0.010)$ was applied.

##### 3.1.3.1 Early Blocks (Blocks 1 and 2)

For Block 1, the main effect of time was significant (*F*_(2, 22)_ = 7.18, *p* = 0.004, *η* ^2^ = 0.39), indicating general task‐related changes across phases. However, the time × stimulation condition interaction was not significant (*F*_(2, 22)_ = 0.45, *p* = 0.643, *η* ^2^ = 0.04), suggesting that these temporal changes were not specific to the stimulation condition. Similarly, in Block 2, no significant time × condition interaction was observed (*F*_(2, 22)_ = 0.32, *p* = 0.727, *η* ^2^ = 0.03). (Table 2).

##### 3.1.3.2 Middle and Late Blocks (Blocks 3–5)

**FIGURE 1:**
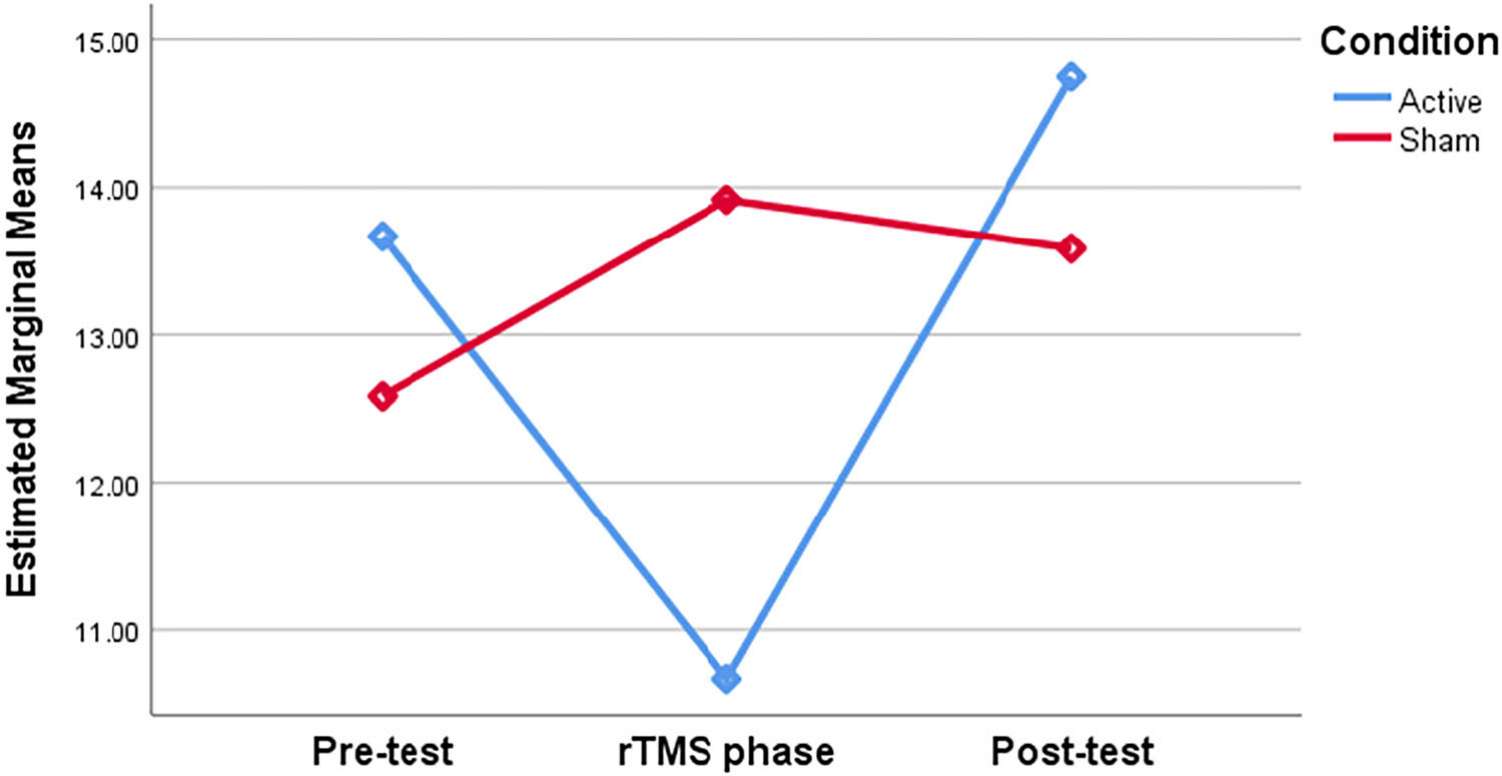
Mean (± SEM) selections from advantageous decks (C and D) in *Block 4* across pretest, rTMS, and posttest phases for active and sham stimulation conditions.

**FIGURE 2:**
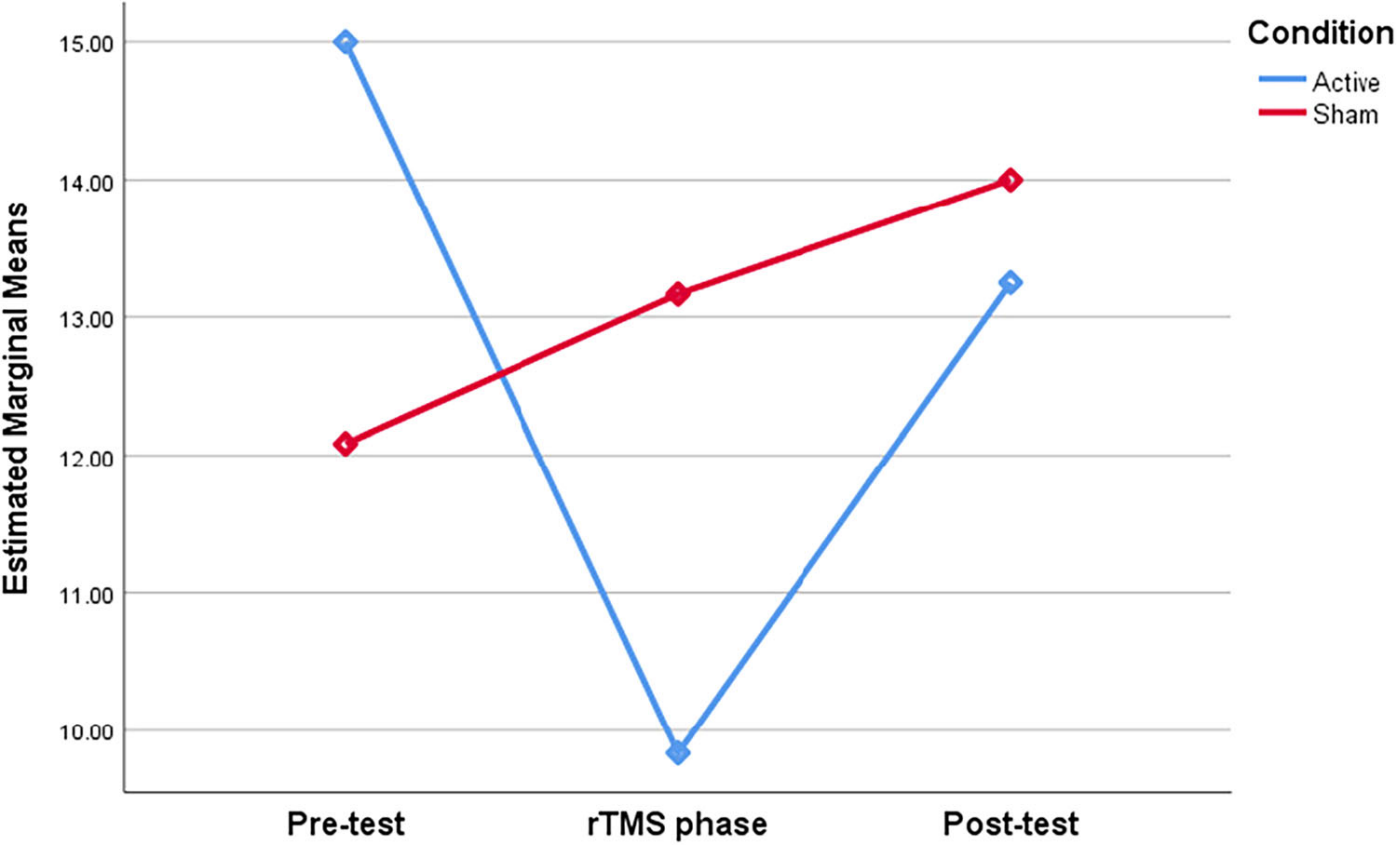
Mean (± SEM) selections from advantageous decks (C and D) in *Block 5* across pretest, rTMS, and posttest phases for active and sham stimulation conditions.

A different pattern emerged during the middle and late stages of the task. The main effect of time for Block 5 (*F*_(2, 22)_ = 5.92, *p* = 0.009, *η* ^2^ = 0.35), and the effect of condition for Blocks 4 (*F*_(1, 11)_ = 9.69, *p* = 0.010, *η* ^2^ = 0.46) and 5 (*F*_(1, 11)_ = 10.10, *p* = 0.009, *η* ^2^ = 0.49), were significant. Significant time × condition interactions were also observed for Block 4 (*F*_(2, 22)_ = 8.18, *p* = 0.002, *η* ^2^ = 0.42) and Block 5 (*F*_(2, 22)_ = 13.87, *p* = 0.0001, *η* ^2^ = 0.56). Given that the univariate analysis with Bonferroni correction ($\frac{\alpha }{5}=0.010)$ indicated significant interactions, the sham and active conditions were subsequently compared across three stages using a dependent samples *t*‐test with Bonferroni correction ($\frac{\alpha }{3}=0.016)$. In Block 4, participants in the active condition selected significantly fewer advantageous cards during the rTMS phase compared with the sham condition (MD = –3.28, *t* = 3.03, SEM = 1.08, *p* = 0.011), whereas no differences emerged at pretest or posttest (*p* > 0.016). A similar pattern was found in Block 5, where rTMS‑phase scores in the active condition were significantly lower than in the sham condition (MD = –3.33, *t* = 3.83, SEM = 0.87, *p* = 0.003), with no significant group differences at pretest or posttest (*p* > 0.016). As illustrated in Figures 1 and 2, advantageous selections during the rTMS phase were consistently lower under active stimulation for Block 4 and Block 5. By contrast, Block 3 alone did not show a significant time × condition interaction (*F*_(2, 22)_ = 0.81, *p* = 0.457, *η* ^2^ = 0.07), after Bonferroni correction (Table 2).

##### 3.1.3.3 Block‐by‐Block Net Score

Block‐by‐block analyses of the IGT Net Score [(C + D) − (A + B)] were conducted to assess how decision‐making dynamics unfolded across the task under active versus sham stimulation. The repeated measure MANOVA was also employed to analyze the eight variables, including the five individual blocks (Blocks 1–5). The results indicated that there is a significant time × condition interaction in the linear combination of five variables (Wilks' Lambda = 0.18, *F*_(2, 10)_ = 6.99, *p* = 0.013, *η* ^2^ = 0.58), whereas the main effects of time (Wilks' Lambda = 0.62, *F*_(2, 10)_ = 1.77, *p* = 0.218, *η* ^2^ = 0.37) and stimulation condition (Wilks' Lambda = 0.57, *F*_(5, 7)_ = 1.02, *p* = 0.421, *η* ^2^ = 0.42) were not significant. Given the significance of the multivariate *F*‐test for time × condition interaction, univariate tests with Bonferroni correction ($\frac{\alpha }{5}=0.01)$ were conducted separately for each of the eight variables. Subsequently, following the Bonferroni correction, no significant effect was observed in any of the blocks or in the overall score: Block 1: (*F*_(2, 22)_ = 0.17, *p* = 0.848, *η* ^2^ = 0.01), Block 2: (*F*_(2, 22)_ = 0.32, *p* = 0.727, *η* ^2^ = 0.03), Block 3: (*F*_(2, 22)_ = 1.29, *p* = 0.318, *η* ^2^ = 0.09), Block 4: (*F*_(2, 22)_ = 2.57, *p* = 0.128, *η* ^2^ = 0.17), and Block 5: (*F*_(2, 22)_ = 4.57, *p* = 0.022, *η* ^2^ = 0.29) (Table 2). Thus, although the multivariate test suggested differences in the overall temporal pattern of performance between stimulation conditions, these effects did not reach statistical significance at the level of individual blocks after correcting for multiple comparisons.

### 3.2 EEG Results

#### 3.2.1 Power of the Theta Frequency

To investigate theta band frequency changes within five ROIs (frontal, central, temporal, parietal, and occipital), a 2 × 2 × 2 repeated measure MANOVA with Bonferroni correction were employed, in which theta frequency band power (with pretest and posttest levels), recording conditions (resting‐state and task‐related, with two levels), and stimulation conditions (real and sham, with two levels) served as within‐subject factors. The results of the repeated measure MANOVA indicated that the main effect of time (Wilks' Lambda = 0.22, *F*_(5, 7)_ = 4.90, *p* = 0.030) and the time × recording condition interaction (Wilks' Lambda = 0.15, *F*_(5, 7)_ = 7.78, *p* = 0.009) were significant for theta power, whereas the effects of stimulation condition (*F*_(5, 7)_ = 0.46, *p* = 0.789), and recording condition (*F*_(5, 7)_ = 2.19, *p* = 0.168), time × stimulation condition (*F*_(5, 7)_ = 0.34, *p* = 0.875), stimulation condition × recording condition (*F*_(5, 7)_ = 2.09, *p* = 0.180), and time × stimulation condition × recording condition (*F*_(5, 7)_ = 0.62, *p* = 0.556) were not significant. Follow‐up univariate analyses with Bonferroni correction ($\frac{\alpha }{5}=0.010)$ for significant effects revealed that the effects of time (*F*_(1, 11)_ = 24.35, *p* = 0.0001, *η* ^2^ = 0.68), and the time × recording condition interaction (*F*_(1, 11)_ = 8.89, *p* = 0.006, *η* ^2^ = 0.44) were significant only for the frontal region. The comparison of theta power between the two recording conditions (rest and task) using dependent‐samples *t*‐test with Bonferroni correction ($\frac{\alpha }{2}=0.025)$ showed that theta power decreased significantly during the task condition compared to the rest condition (MD = 0.09, *t* = 4.50, SEM = 0.02, *p* = 0.001).

#### 3.2.2 Power of Beta Frequency

The results of 2 × 2 × 2 repeated measure MANOVA for five ROIs indicated that among the main and interaction effects, only the interaction effect of time × stimulation condition (Wilks' Lambda = 0.14, *F*_(5, 7)_ = 8.65, *p* = 0.007) and the main effect of time (Wilks' Lambda = 0.11, *F*_(5, 7)_ = 11.02, *p* = 0.004) were significant. While the effects of stimulation condition (*F*_(5, 7)_ = 3.12, *p* = 0.083), recording condition (*F*_(5, 7)_ = 2.56, *p* = 0.131), time × recording condition (*F*_(5, 7)_ = 0.32, *p* = 0.883), stimulation condition × recording condition (*F*_(5, 7)_ = 1.59, *p* = 0.282), and time × stimulation condition × recording condition (*F*_(5, 7)_ = 2.03, *p* = 0.186) were not significant. The follow‐up analyses using univariate *F* with Bonferroni correction ($\frac{\alpha }{5}=0.010)$ indicated that the main effect of time and time × stimulation condition interaction were significant only in the frontal (time: *F*_(1, 11)_ = 13.52, *p* = 0.004; time × stimulation: *F*_(1, 11)_ = 14.67, *p* = 0.003) and temporal regions (time: *F*_(1, 11)_ = 11.12, *p* = 0.007; time × stimulation: *F*_(1, 11)_ = 10.69, *p* = 0.007). The subsequent dependent samples *t*‐tests with Bonferroni correction ($\frac{\alpha }{2}=0.025)$ for both the frontal (MD = −0.16, *t* = 3.17, SEM = 0.05, *p* = 0.009) and temporal (MD = −0.09, *t* = 3.0, SEM = 0.03, *p* = 0.012) regions revealed that beta power increased in the posttest phase in the active stimulation condition compared to the sham condition. No differences were observed between the two stimulation conditions for either region at pretest (*p* > 0.025).

#### 3.2.3 Frontal Alpha Asymmetry

A 2 × 2 × 2 repeated‐measures MANOVA was performed to examine FAA. Relative asymmetry values derived from the F4–F3 and F8–F7 electrode pairs, as well as absolute asymmetry values derived from the same F4–F3 and F8–F7 regions, were entered as dependent variables into the model. The results of the multivariate *F* showed that the main effects of time (Wilks' Lambda = 0.27, *F*_(4, 8)_ = 5.28, *p* = 0.022) and recording condition (Wilks' Lambda = 0.29, *F*_(4, 8)_ = 4.94, *p* = 0.026), as well as the time × recording condition (Wilks' Lambda = 0.22, *F*_(4, 8)_ = 7.08, *p* = 0.010), were statistically significant. Therefore, univariate repeated measures ANOVA with Bonferroni correction ($\frac{\alpha }{4}=0.0125)$ were conducted to determine in which type of asymmetry (relative and absolute) and in which region (F4–F3 and F8–F7) the differences exist. In this regard, the results showed that the main effect of time (*F*_(1, 11)_ = 13.54, *p* = 0.004) was significant for the relative asymmetry of F4–F3, and the main effect of recording condition *F*_(1, 11)_ = 10.21, *p* = 0.009) and time × recording condition (*F*_(1, 11)_ = 15.58, *p* = 0.002) were significant for the absolute asymmetry of F8–F7. Finally, after applying the Bonferroni correction ($\frac{\alpha }{4}=0.0125)$, only the main effect of time remained significant for the relative asymmetry of F4–F3 (*p* < 0.0125). The effects of stimulation condition (*F*_(4, 8)_ = 0.82, *p* = 0.784) time × stimulation condition (*F*_(4, 8)_ = 1.07, *p* = 0.431), stimulation condition × recording condition (*F*_(4, 8)_ = 0.48, *p* = 0.751), and time × stimulation condition × recording condition (*F*_(4, 8)_ = 3.44, *p* = 0.069) were also not significant.

#### 3.2.4 Theta/Beta Ratio

A 2 × 2 × 2 repeated measure MANOVA was conducted on TBR across five ROIs. The multivariate tests showed no significant main effects of time (*F*_(5, 7)_ = 2.39, *p* = 0.161) or stimulation condition (*F*_(5,7)_ = 1.23, *p* = 0.387), and no significant effects of recording condition (*F*_(5,7)_ = 1.29, *p* = 0.365). Furthermore, none of the time × stimulation condition (*F*_(5, 7)_ = 0.83, *p* = 0.564), time × recording condition (*F*_(5, 7)_ = 0.23, *p* = 0.937), or time × stimulation condition × recording condition (*F*_(5, 7)_ = 0.57, *p* = 0.718) effects were significant. The only significant interaction effect was that of stimulation condition × recording condition (Wilks' Lambda = 0.15, *F*_(5, 7)_ = 7.69, *p* = 0.009). Therefore, the univariate *F*‐tests with Bonferroni correction ($\frac{\alpha }{5}=0.010)$ were conducted only for stimulation condition × recording condition. The results indicated that the TBR was significant only in the frontal region (*F*_(5, 7)_ = 8.99, *p* = 0.006) and was not significant in other regions (*p* > 0.010). Finally, the paired‐samples *t*‐tests with Bonferroni correction were performed for each condition of stimulation and recording (sham–rest, sham–task, active–rest, active–task). The results showed that only the difference in the sham stimulation condition under the task condition was significant (MD = −0.31, *t* = 7.68, SEM = 0.04, *p* = 0.001). In other words, for sham stimulation under the task condition, the TBR in the frontal region showed a significant decrease from pretest to posttest. Taken together, TBR was not modulated by rTMS; the observed change was restricted to a sham‐‑task decrease in frontal TBR, likely reflecting task‑related or fatigue‑related influences rather than stimulation‑induced effects.

## 4 Discussion

The present study provides preliminary evidence for the involvement of the right DLPFC in adaptive decision‑making. High‑frequency (20 Hz) rTMS delivered during performance of the IGT produced a transient reduction in advantageous choices (C + D) during the stimulation phase, with behavioral performance returning to baseline following stimulation. This pattern is consistent with a temporary interference effect of online right‐DLPFC stimulation on value‐integration processes underlying decision‐making.

In contrast, post‑stimulation EEG analyses revealed increased β‐band power in frontal and temporal regions during task performance. Because EEG was not recorded during stimulation, these findings are limited to post‐intervention measurements and do not allow inferences regarding neural dynamics during stimulation. Importantly, no stimulation‐related effects were observed for TBR or FAA, indicating that these measures were not modulated by right‐DLPFC rTMS in the present sample.

Overall, the results indicate that behavioral effects were transient and restricted to the stimulation period, whereas electrophysiological changes were limited to post‐stimulation β‐band activity. These effects were not uniformly expressed across EEG indices, but were primarily observed in β‐band power. However, given the small sample size and exploratory nature of the analyses, these findings should be interpreted cautiously.

### 4.1 Selective Disruption of Advantageous, Feedback‐Based Decision‐Making

The reduction in advantageous deck selections observed during the stimulation phase, followed by a return to baseline after stimulation, suggests a transient and selective disruption of feedback‑based decision‑making induced by high‑frequency (20 Hz) rTMS applied to the right DLPFC. The IGT assesses the ability to learn from feedback and gradually shift from disadvantageous to advantageous choices, with performance reflecting sensitivity to long‑term reward contingencies (Bechara et al. 1999). The selective reduction in advantageous choices observed here suggests that rTMS may have transiently influenced the integration of outcome history necessary for adaptive decision‑making. Notably, the reduction in advantageous choices was observed specifically in Blocks 4 and 5 during the stimulation phase, whereas no significant stimulation‐related differences were detected in the earlier blocks.

This finding aligns with the established role of the prefrontal cortex in evaluating long‑term consequences and guiding behavior based on cumulative feedback. Classical lesion studies have shown that dysfunction in prefrontal regions, particularly the ventromedial and dorsolateral prefrontal cortices, impairs the ability to incorporate future outcomes into ongoing decisions (Bechara 2000; Fellows 2005). In our paradigm, the decline was specific to the active rTMS condition and was confined to the stimulation phase, indicating that high‑frequency stimulation delivered online during task execution may transiently interfere with ongoing cognitive operations in a state‐dependent manner.

Although high‑frequency rTMS is often characterized as excitatory in offline paradigms, its impact during ongoing cognitive processing can be state‑dependent and may have disruptive or facilitatory effects depending on task context. Online stimulation may have induced transient neural noise within task‑relevant networks, potentially affecting the computations required for integrating feedback and guiding advantageous choices (Beynel et al. 2019). Related work has shown that inhibitory stimulation of the right DLPFC modulates risk‑related and impulsive decision‑making (Knoch et al. 2006; Tulviste and Bachmann 2019) and influences delay‑discounting behavior (Cho et al. 2010; Cho et al. 2012). In contrast to inhibitory protocols that often increase selection of high‑risk options, our online high‑frequency rTMS selectively reduced advantageous (C and D) choices without increasing disadvantageous (A and B) selections. This pattern is consistent with a transient disruption of long‑term value integration and strategic adjustment processes, while more immediate loss‑avoidance tendencies may have remained relatively unaffected. The rapid normalization of performance after stimulation is compatible with the possibility that online rTMS induced transient perturbations within task‐relevant networks, which may have contributed to momentary interference followed by rapid behavioral recovery.

### 4.2 EEG Correlates: Post‐Stimulation β‐Band Power Increases

Post‑stimulation analyses revealed a significant increase in β‑band power during task performance within the frontal and temporal regions. Given that the EEG was recorded in separate pre‐ and post‐stimulation task sessions (and not during stimulation), these findings should be interpreted specifically as post‐intervention neurophysiological changes rather than online effects. Beta oscillations (13–30 Hz) have been implicated in top‑down cognitive control, attentional maintenance, and the integration of sensory–motor information during goal‑directed behavior (Engel and Fries 2010; Spitzer and Haegens 2017). Consequently, the post‑rTMS enhancement of β power may reflect an altered cortical state or state‐dependent neurophysiological following transient stimulation‐induced disruption, potentially suggesting that neurophysiological modulation may extend beyond the transient behavioral impairment observed during the stimulation phase, although this interpretation remains indirect given the absence of EEG during stimulation.

The spatial distribution of β enhancement across frontal and temporal regions is broadly consistent with prior TMS neuroimaging findings demonstrating that focal stimulation can induce changes in functionally connected regions (Bestmann et al. 2008; Ruff et al. 2009; Siebner et al. 2009). While such patterns may reflect context‐dependent modulation of distributed networks, the present data do not provide direct evidence for large‐scale or sustained network reorganization. Accordingly, the observed effects are more cautiously interpreted as localized post‐stimulation changes in task‐related oscillatory activity, potentially consistent with transient functional redistribution within task‐relevant circuits.

Single‑session rTMS can produce short‑lived β after‑effects depending on stimulation parameters, although such oscillatory changes do not necessarily co‐vary with behavioral effects. This dissociation is reflected in our data, where behavioral performance returned to baseline after stimulation, while a significant increase in β power was observed in the post‐stimulation task session, suggesting that EEG measures may capture neurophysiological changes that are not directly expressed in overt behavior (Thut and Pascual‐Leone 2010). In the present study, behavioral performance rapidly returned toward baseline despite the presence of increased post‐stimulation β power, suggesting a possible temporal dissociation between transient behavioral disruption and ongoing neurophysiological modulation (Hartwigsen and Volz 2021; O'Shea et al. 2007; Zanto et al. 2013). However, because the EEG was not recorded during stimulation, no inferences can be made regarding neural dynamics during the intervention, and this dissociation should be interpreted strictly in terms of pre‐ and post‐stimulation measurements.

These findings may be interpreted in a cautious, descriptive manner rather than within a strict mechanistic phase‐based framework. Specifically, the results are consistent with the possibility that (i) high‐frequency rTMS transiently affected right‐DLPFC‐dependent processing during task execution, as suggested by state‐dependent models of online TMS, which emphasize the interaction between stimulation effects and ongoing cognitive activity (Siebner et al. 2009; Silvanto and Cattaneo 2017; Silvanto et al. 2008); and (ii) post‐stimulation β increases reflect short‐term changes in cortical oscillatory state during subsequent task performance. In this context, the observed post‐stimulation modulation of β activity may reflect a temporary adjustment of task‐related neural dynamics following perturbation, although the absence of EEG recordings during stimulation precludes direct inferences regarding online neural mechanisms. Finally, the selective modulation of β activity—specifically its increase during post‐stimulation task performance following active stimulation—together with the absence of stimulation effects on TBR and FAA, supports the interpretation that right‐DLPFC rTMS produces frequency‐specific neurophysiological effects rather than broad alterations in attentional or motivational asymmetry. Importantly, given the small sample size (*N* = 12) and the exploratory nature of the analyses, the interpretation of EEG‐based neurophysiological changes should be considered preliminary and hypothesis‐generating rather than confirmatory.

### 4.3 Frontal Theta Dynamics: Task‑Dependent and Region‑Specific Modulations

Analyses of theta‐band activity across the five ROIs revealed a significant main effect of time and a time × recording condition interaction, driven specifically by changes in the frontal region. Post hoc comparisons showed that frontal theta power decreased from pre‑ to post‑measurement during task performance, whereas no significant temporal change was observed at rest. The region‐specific nature of this effect is broadly consistent with the frontal midline theta (FMT) signal, which has been linked to medial prefrontal and anterior cingulate cortex activity (Cavanagh and Shackman 2015; Cohen 2011). This pattern of reduced frontal theta during task engagement is consistent with prior work suggesting that FMT is sensitive to variations in cognitive control demands and performance monitoring processes. In particular, frontal theta has been shown to increase during high‐conflict, error‐related, or uncertain decision contexts, and to vary with the level of ongoing monitoring requirements (Cavanagh et al. 2012; Cavanagh and Frank 2014; Cohen 2011; Cohen and Donner 2013). Accordingly, the present reduction in theta power may reflect a task‐dependent shift in the level of cognitive control engagement during task execution. The absence of significant effects in central, temporal, parietal, and occipital regions further supports the selective involvement of frontal theta dynamics in task‐related modulation. Importantly, no stimulation‐related effects were observed for theta activity, suggesting that frontal theta dynamics remained stable across active and sham rTMS conditions in the present sample. Taken together, these findings indicate that frontal theta activity is primarily modulated by task context rather than by stimulation condition, and that the observed effects reflect a region‐specific and frequency‐specific pattern of neural modulation. Overall, this pattern is consistent with a frequency‐specific dissociation, wherein β‐band dynamics were modulated following right‐DLPFC rTMS, whereas frontal theta activity did not show detectable stimulation‐related changes in the present sample.

### 4.4 Time‐Dependent but Non‐Stimulation‐Related Effects in TBR and FAA

#### 4.4.1 Theta/Beta Ratio

The TBR has been proposed as an electrophysiological index of attentional and executive control processes, with lower values typically reflecting more efficient top‑down regulation and sustained cognitive engagement (Angelidis et al. 2016; Putman et al. 2014; D. Schutter and Kenemans 2022). In the present study, no significant main effects or interactions involving time or stimulation condition were observed. The only significant finding was a localized stimulation × recording condition interaction in the frontal region, which follow‐up analyses revealed to be driven exclusively by a reduction in frontal TBR during the sham–task condition.

Because this effect was restricted to the sham condition and no stimulation‐related differences were observed, the findings do not provide evidence that right‐DLPFC rTMS modulated TBR. The observed reduction in frontal TBR during sham–task performance may reflect nonspecific task familiarity or practice‐related influences rather than stimulation‐related modulation. Similar reductions in frontal theta activity have been reported as task demands become more familiar or predictable (Wong, Chan, and Mak 2014)—a pattern broadly consistent with the neural efficiency hypothesis, whereby reduced theta activity has been proposed to reflect automatization and diminished reliance on cognitive control (Huycke et al. 2021; Margraf et al. 2023). However, given the absence of a direct manipulation of familiarity, this interpretation remains speculative.

Overall, the absence of stimulation‐related effects on TBR, despite the observed post‐stimulation increase in β power, further suggests that the electrophysiological effects of rTMS were frequency‐specific rather than reflected in broader ratio‐based indices of executive control. However, interpretations of TBR findings should remain cautious given the indirect nature of ratio measures and the limited sample size.

#### 4.4.2 Frontal Alpha Asymmetry

FAA has been widely associated with individual differences in affective and motivational processing, with relatively reduced left‑frontal alpha power typically interpreted as greater approach‑oriented tendencies (Davidson 1998; Harmon‐Jones et al. 2010). In the present study, although multivariate analyses initially revealed significant effects involving Time and Recording Condition, only the main effect of Time on relative F4–F3 asymmetry remained significant following Bonferroni correction. Importantly, no effects involving stimulation condition were observed at either the multivariate or univariate level, indicating that active 20‐Hz right‐DLPFC rTMS did not significantly modulate FAA.

The observed effect of Time on relative F4–F3 asymmetry suggests that FAA exhibited some degree of temporal variability across the experimental session. Such changes may reflect fluctuations in cognitive engagement, arousal, or other state‐dependent factors that evolved over time. However, because the present design was not intended to isolate the specific processes underlying these temporal changes, any interpretation regarding their functional significance should remain tentative.

The absence of FAA modulation following active rTMS suggests that FAA may be relatively insensitive to the acute effects of this stimulation protocol. To our knowledge, relatively few studies have specifically examined the immediate effects of single‐session DLPFC rTMS on FAA in healthy individuals, making direct comparisons with the present findings difficult. Nevertheless, Möbius et al. (2017) similarly reported no significant changes in FAA following a single session of 10‐Hz rTMS over the left DLPFC in healthy participants (Möbius et al. 2017). Together with the present findings, these results suggest that FAA may be relatively insensitive to the acute effects of a single session of prefrontal rTMS.

Interestingly, findings from multi‐session rTMS studies in depression have also been mixed. For example, Spronk et al. (2008) observed no significant change in FAA despite clinical improvement following 20 sessions of left‐DLPFC rTMS (Spronk et al. 2008). Similarly, Li et al. (2013) found that, following a 2‐week course of left‐DLPFC rTMS, FAA changes failed to differentiate treatment‐resistant depression patients from healthy controls and did not distinguish treatment responders from nonresponders (Li et al. 2013). Although these studies involved clinical populations and substantially different stimulation protocols, they further suggest that FAA may not consistently reflect rTMS‐induced neurophysiological or clinical changes.

At the same time, the absence of stimulation‐related effects should be interpreted cautiously. While the present findings, together with previous evidence, may suggest that FAA may be relatively trait‐like in some contexts rather than strongly state‐dependent following single‐session DLPFC stimulation, such an interpretation remains tentative, particularly given that the present study was conducted in healthy individuals who were not characterized by marked baseline abnormalities in frontal activity.

Overall, the lack of FAA modulation, together with the null findings for TBR and the selective increase in β power following stimulation, is consistent with the conclusion that the electrophysiological effects of right‐DLPFC rTMS were not uniformly expressed across EEG measures. Rather, stimulation‐related effects appeared to be more evident in β‐band activity than in asymmetry‐ or ratio‐based indices such as FAA and TBR.

### 4.5 Limitations and Future Directions

Several methodological considerations merit discussion. First, the absence of EEG recording during the intervention phase precludes direct correlation between acute behavioral impairment and underlying neural dynamics during stimulation. We cannot determine whether the online disruption reflects excessive beta entrainment, theta suppression, disrupted cross‐frequency coupling, or fundamentally different oscillatory dynamics. Future studies employing concurrent TMS‐EEG with advanced artifact suppression techniques (e.g., sample‐and‐hold circuits, source reconstruction) are needed to directly link trial‐by‐trial neural perturbations to decision‐making deficits.

Second, our sample (*N* = 12) limits statistical power, increases uncertainty around effect‐size estimation, and restricts the ability to examine individual differences in responsiveness to stimulation. Although comparable sample sizes are common in mechanistic TMS‐EEG studies (typical *N* = 10–15; Veniero et al. 2011), the limited sample size should be considered a central constraint when interpreting the present findings. The observed group‐level impairment may mask substantial interindividual variability influenced by cortical anatomy, baseline neurophysiology, and cortical excitability. Larger cohorts with baseline neurophysiological and structural characterization (e.g., TBR, TMS‐evoked potentials, diffusion tensor imaging) could help identify factors associated with differential responsiveness to stimulation. Although several effects survived correction for multiple comparisons, replication in larger samples will be essential to establish the robustness and generalizability of the reported findings.

Third, targeting relied on the standardized 10–20 EEG system rather than MRI‐based neuronavigation, introducing potential interindividual variability in the precise location and intensity of the induced electric field. Future studies utilizing frameless stereotaxic neuronavigation or electric field modeling could achieve more anatomically precise targeting.

Fourth, our EEG analysis was hypothesis‐driven, focusing on specific frequency bands and electrode sites. While this provided clear insights and reduced multiple comparisons, it may not have captured the full scope of rTMS‐induced network dynamics. Whole‐brain approaches—such as source localization, graph‐theoretic connectivity analysis, or machine learning classification— may reveal distributed oscillatory changes and large‐scale network reconfigurations (Siebner et al. 2009). Despite these limitations, our findings provide preliminary evidence consistent with a state‐dependent involvement of the right DLPFC in risky decision‐making, although this interpretation should be considered in light of the limited sample size and exploratory nature of several analyses.

Finally, another limitation of the present study relates to the interpretation of band‐limited EEG power. The spectral analysis employed here does not explicitly separate periodic (oscillatory) activity from the aperiodic (1/*f*‐like) component of the EEG signal. As a result, the observed changes in frequency‐specific power may reflect not only modulation of oscillatory processes but also shifts in the underlying aperiodic structure, which has been linked to changes in cortical excitation–inhibition balance. Future studies could address this limitation by applying spectral parameterization approaches (e.g., FOOOF/specparam) (Donoghue et al. 2020) to disentangle oscillatory and aperiodic contributions, thereby providing a more precise characterization of the neurophysiological mechanisms underlying the observed effects.

Future work should prioritize the following: (1) concurrent TMS‐EEG to capture online neural dynamics; (2) neuronavigated targeting to reduce spatial variability; (3) whole‐brain network analyses to elucidate distributed mechanisms; and (4) real‐time EEG‐triggered rTMS to synchronize stimulation with specific oscillatory phases, potentially enhancing mechanistic precision. Longitudinal studies tracking beta power changes across repeated stimulation sessions may help determine whether these neurophysiological effects generalize to clinically relevant populations characterized by maladaptive decision‐making. Integration of rTMS with functional neuroimaging could clarify connectivity changes and elucidate network‐level compensatory processes. These advances will be critical for translating mechanistic insights into precision neuromodulation protocols.

## 5 Conclusion

In summary, high‑frequency (20 Hz) rTMS applied to the right DLPFC produced a selective and transient impairment in advantageous decision‑making during performance of the IGT, while post‑stimulation EEG analyses revealed increases in β‑band power within frontal and temporal regions. This dissociation between short‑lived behavioral disruption and post‑stimulation oscillatory modulation suggests that online perturbation of the right DLPFC may transiently interfere with value‑integration processes and may be associated with neurophysiological adjustments within distributed cortical networks.

The absence of stimulation effects on frontal theta activity, TBR, and FAA is consistent with the possibility that the observed modulation was relatively frequency‑specific rather than reflecting broad alterations in motivational or attentional states. Instead, the findings are consistent with a potential involvement of right‑DLPFC stimulation in executive processes supporting feedback integration and adaptive decision‑making.

Together, these results provide preliminary evidence consistent with a role of the right DLPFC to value integration during adaptive decision‐making and suggest that online rTMS may produce temporally dissociable behavioral and neurophysiological effects. However, these findings should be interpreted in light of the limited sample size and require replication in larger samples.

## Author Contributions

**Reza Kazemi**: conceptualization, methodology, project administration, writing – original draft preparation. **Reza Rostami**: supervision, writing – original draft preparation. **Maryam Majidinezhad**: formal analysis, writing – original draft preparation., **Hamed Hosseini Zarrabi**: writing – original draft preparation. **Sanaz Khomami**: project administration, writing – review and editing. **Mehdi Rezaei**: formal analysis, writing – original draft. **Ahmad Zandbagleh**: formal analysis, writing – original draft preparation.

## Funding

The authors have nothing to report.

## Disclosure

The authors have nothing to report.

## Conflicts of Interest

The authors declare no conflicts of interest.

## Data Availability

All data are included in the manuscript. The datasets analyzed here are available from the corresponding author on reasonable request.

## References

[brb371584-bib-0001] Adida, M. , F. Jollant , L. Clark , et al. 2011. “Trait‐Related Decision‐making Impairment in the Three Phases of Bipolar Disorder.” Biological Psychiatry 70, no. 4: 357–365. 10.1016/j.biopsych.2011.01.018. 21429477

[brb371584-bib-0002] Ahn, H. M. , S. E. Kim , and S. H. Kim . 2013. “The Effects of High‐Frequency rTMS over the Left Dorsolateral Prefrontal Cortex on Reward Responsiveness.” Brain Stimulation 6, no. 3: 310–314. 10.1016/j.brs.2012.05.013. 22749028

[brb371584-bib-0003] Angelidis, A. , W. van der Does , L. Schakel , and P. Putman . 2016. “Frontal EEG Theta/Beta Ratio as an Electrophysiological Marker for Attentional Control and Its Test‐Retest Reliability.” Biological Psychology 121: 49–52. 10.1016/j.biopsycho.2016.09.008. 27697551

[brb371584-bib-0004] Bechara, A. , A. R. Damasio , H. Damasio , and S. W. Anderson . 1994. “Insensitivity to Future Consequences Following Damage to Human Prefrontal Cortex.” Cognition 50, no. 1–3: 7–15. 10.1016/0010-0277(94)90018-3. 8039375

[brb371584-bib-0005] Bechara, A. 2000. “Emotion, Decision Making and the Orbitofrontal Cortex.” Cerebral Cortex 10, no. 3: 295–307. 10.1093/cercor/10.3.295. 10731224

[brb371584-bib-0006] Bechara, A. , H. Damasio , A. R. Damasio , and G. P. Lee . 1999. “Different Contributions of the Human Amygdala and Ventromedial Prefrontal Cortex to Decision‐Making.” Journal of Neuroscience 19, no. 13: 5473–5481. 10.1523/JNEUROSCI.19-13-05473.1999. 10377356 PMC6782338

[brb371584-bib-0007] Bestmann, S. , O. Swayne , F. Blankenburg , et al. 2008. “Dorsal Premotor Cortex Exerts State‐Dependent Causal Influences on Activity in Contralateral Primary Motor and Dorsal Premotor Cortex.” Cerebral Cortex 18, no. 6: 1281–1291. 10.1093/cercor/bhm159. 17965128 PMC2600427

[brb371584-bib-0008] Beynel, L. , L. G. Appelbaum , B. Luber , et al. 2019. “Effects of Online Repetitive Transcranial Magnetic Stimulation (rTMS) on Cognitive Processing: A Meta‐Analysis and Recommendations for Future Studies.” Neuroscience & Biobehavioral Reviews 107: 47–58. 10.1016/j.neubiorev.2019.08.018. 31473301 PMC7654714

[brb371584-bib-0009] Bowman, H. , J. L. Brooks , O. Hajilou , A. Zoumpoulaki , and V. Litvak . 2020. “Breaking the Circularity in Circular Analyses: Simulations and Formal Treatment of the Flattened Average Approach.” PLoS Computational Biology 16, no. 11: e1008286. 10.1371/journal.pcbi.1008286. 33226982 PMC7721178

[brb371584-bib-0010] Cavanagh, J. F. , C. M. Figueroa , M. X. Cohen , and M. J. Frank . 2012. “Frontal Theta Reflects Uncertainty and Unexpectedness During Exploration and Exploitation.” Cerebral Cortex 22, no. 11: 2575–2586. 10.1093/cercor/bhr332. 22120491 PMC4296208

[brb371584-bib-0011] Cavanagh, J. F. , and M. J. Frank . 2014. “Frontal Theta as a Mechanism for Cognitive Control.” Trends in Cognitive Sciences 18, no. 8: 414–421. 10.1016/j.tics.2014.04.012. 24835663 PMC4112145

[brb371584-bib-0012] Cavanagh, J. F. , and A. J. Shackman . 2015. “Frontal Midline Theta Reflects Anxiety and Cognitive Control: Meta‐Analytic Evidence.” Journal of Physiology 109, no. 1–3: 3–15. 10.1016/j.jphysparis.2014.04.003. 24787485 PMC4213310

[brb371584-bib-0013] Chen, S. , P. Yang , T. Chen , H. Su , H. Jiang , and M. Zhao . 2020. “Risky Decision‐Making in Individuals With Substance Use Disorder: A Meta‐Analysis and Meta‐Regression Review.” Psychopharmacology 237, no. 7: 1893–1908. 10.1007/s00213-020-05506-y. 32363438

[brb371584-bib-0014] Cho, S. S. , J. H. Ko , G. Pellecchia , T. Van Eimeren , R. Cilia , and A. P. Strafella . 2010. “Continuous Theta Burst Stimulation of Right Dorsolateral Prefrontal Cortex Induces Changes in Impulsivity Level.” Brain Stimulation 3, no. 3: 170–176. 10.1016/j.brs.2009.10.002. 20633446 PMC3707839

[brb371584-bib-0015] Cho, S. S. , G. Pellecchia , J. H. Ko , et al. 2012. “Effect of Continuous Theta Burst Stimulation of the Right Dorsolateral Prefrontal Cortex on Cerebral Blood Flow Changes During Decision Making.” Brain Stimulation 5, no. 2: 116–123. 10.1016/j.brs.2012.03.007. 22494829 PMC3707841

[brb371584-bib-0016] Coan, J. A. , and J. J. Allen . 2004. “Frontal EEG Asymmetry as a Moderator and Mediator of Emotion.” Biological Psychology 67, no. 1–2: 7–50. 10.1016/j.biopsycho.2004.03.002. 15130524

[brb371584-bib-0017] Cohen, M. X. 2011. “Error‐Related Medial Frontal Theta Activity Predicts Cingulate‐Related Structural Connectivity.” Neuroimage 55, no. 3: 1373–1383. 10.1016/j.neuroimage.2010.12.072. 21195774

[brb371584-bib-0018] Cohen, M. X. , and T. H. Donner . 2013. “Midfrontal Conflict‐Related Theta‐Band Power Reflects Neural Oscillations That Predict Behavior.” Journal of Neurophysiology 110, no. 12: 2752–2763. 10.1152/jn.00479.2013. 24068756

[brb371584-bib-0019] Davidson, R. J. 1992. “Anterior Cerebral Asymmetry and the Nature of Emotion.” Brain and Cognition 20, no. 1: 125–151. 10.1016/0278-2626(92)90065-T. 1389117

[brb371584-bib-0020] Davidson, R. J. 1998. “Affective Style and Affective Disorders: Perspectives From Affective Neuroscience.” Cognition & Emotion 12, no. 3: 307–330. 10.1080/026999398379628.

[brb371584-bib-0021] Derogatis, L. R. , R. S. Lipman , and L. Covi . 1973. “SCL‐90: An Outpatient Psychiatric Rating Scale–Preliminary Report.” Psychopharmacology Bulletin 9, no. 1: 13–28. 4682398

[brb371584-bib-0022] Donoghue, T. , M. Haller , E. J. Peterson , et al. 2020. “Parameterizing Neural Power Spectra Into Periodic and Aperiodic Components.” Nature Neuroscience 23, no. 12: 1655–1665. 10.1038/s41593-020-00744-x. 33230329 PMC8106550

[brb371584-bib-0023] Engel, A. K. , and P. Fries . 2010. “Beta‐Band Oscillations—Signalling the Status Quo?” Current Opinion in Neurobiology 20, no. 2: 156–165. 10.1016/j.conb.2010.02.015. 20359884

[brb371584-bib-0024] Ernst, M. , and M. P. Paulus . 2005. “Neurobiology of Decision Making: A Selective Review From a Neurocognitive and Clinical Perspective.” Biological Psychiatry 58, no. 8: 597–604. 10.1016/j.biopsych.2005.06.004. 16095567

[brb371584-bib-0025] Fellows, L. K. 2005. “Different Underlying Impairments in Decision‐Making Following Ventromedial and Dorsolateral Frontal Lobe Damage in Humans.” Cerebral Cortex 15, no. 1: 58–63. 10.1093/cercor/bhh108. 15217900

[brb371584-bib-0026] Figner, B. , D. Knoch , E. J. Johnson , et al. 2010. “Lateral Prefrontal Cortex and Self‐Control in Intertemporal Choice.” Nature Neuroscience 13, no. 5: 538–539. 10.1038/nn.2516. 20348919

[brb371584-bib-0027] Fond, G. , S. Bayard , D. Capdevielle , et al. 2013. “A Further Evaluation of Decision‐Making Under Risk and Under Ambiguity in Schizophrenia.” European Archives of Psychiatry and Clinical Neuroscience 263, no. 3: 249–257. 10.1007/s00406-012-0330-y. 22639243

[brb371584-bib-0028] Gianotti, L. R. R. , D. Knoch , P. L. Faber , et al. 2009. “Tonic Activity Level in the Right Prefrontal Cortex Predicts Individuals' Risk Taking.” Psychological Science 20, no. 1: 33–38. 10.1111/j.1467-9280.2008.02260.x. 19152538

[brb371584-bib-0029] Harmon‐Jones, E. , and J. J. Allen . 1997. “Behavioral Activation Sensitivity and Resting Frontal EEG Asymmetry: Covariation of Putative Indicators Related to Risk for Mood Disorders.” Journal of Abnormal Psychology 106, no. 1: 159–163. 10.1037/0021-843X.106.1.159. 9103728

[brb371584-bib-0030] Harmon‐Jones, E. , P. A. Gable , and C. K. Peterson . 2010. “The Role of Asymmetric Frontal Cortical Activity in Emotion‐Related Phenomena: A Review and Update.” Biological Psychology 84, no. 3: 451–462. 10.1016/j.biopsycho.2009.08.010. 19733618

[brb371584-bib-0031] Hartwigsen, G. , and L. J. Volz . 2021. “Probing Rapid Network Reorganization of Motor and Language Functions via Neuromodulation and Neuroimaging.” Neuroimage 224: 117449. 10.1016/j.neuroimage.2020.117449. 33059054

[brb371584-bib-0032] Huycke, P. , P. Verbeke , C. N. Boehler , and T. Verguts . 2021. “Theta and Alpha Power Across Fast and Slow Timescales in Cognitive Control.” European Journal of Neuroscience 54, no. 2: 4581–4594. 10.1111/ejn.15320. 34033152

[brb371584-bib-0033] Knoch, D. , L. R. Gianotti , A. Pascual‐Leone , et al. 2006. “Disruption of Right Prefrontal Cortex by Low‐Frequency Repetitive Transcranial Magnetic Stimulation Induces Risk‐Taking Behavior.” Journal of Neuroscience 26, no. 24: 6469–6472. 10.1523/JNEUROSCI.0804-06.2006. 16775134 PMC6674035

[brb371584-bib-0034] Kovács, I. , M. J. Richman , Z. Janka , A. Maraz , and B. Andó . 2017. “Decision Making Measured by the Iowa Gambling Task in Alcohol Use Disorder and Gambling Disorder: A Systematic Review and Meta‐Analysis.” Drug and Alcohol Dependence 181: 152–161. 10.1016/j.drugalcdep.2017.09.023. 29055269

[brb371584-bib-0035] Li, C.‐T. , L.‐F. Chen , P.‐C. Tu , et al. 2013. “Impaired Prefronto‐Thalamic Functional Connectivity as a Key Feature of Treatment‐Resistant Depression: A Combined MEG, PET and rTMS Study.” PLoS ONE 8, no. 8: e70089. 10.1371/journal.pone.0070089. 23936378 PMC3732278

[brb371584-bib-0036] Margraf, L. , D. Krause , and M. Weigelt . 2023. “Frontal Theta Reveals Further Information About Neural Valence‐Dependent Processing of Augmented Feedback in Extensive Motor Practice—A Secondary Analysis.” European Journal of Neuroscience 57, no. 8: 1297–1316. 10.1111/ejn.15951. 36878863

[brb371584-bib-0037] Miller, E. K. , and J. D. Cohen . 2001. “An Integrative Theory of Prefrontal Cortex Function.” Annual Review of Neuroscience 24, no. 1: 167–202. 10.1146/annurev.neuro.24.1.167. 11283309

[brb371584-bib-0038] Möbius, M. , L. Lacomble , T. Meyer , et al. 2017. “Repetitive Transcranial Magnetic Stimulation Modulates the Impact of a Negative Mood Induction.” Social Cognitive and Affective Neuroscience 12, no. 4: 526–533. 28008080 10.1093/scan/nsw180 PMC5390712

[brb371584-bib-0039] Nisticò, V. , A. De Angelis , R. Erro , B. Demartini , and L. Ricciardi . 2021. “Obsessive‐Compulsive Disorder and Decision Making Under Ambiguity: A Systematic Review With Meta‐Analysis.” Brain Sciences 11, no. 2: 143. 10.3390/brainsci11020143. 33499211 PMC7912249

[brb371584-bib-0040] O'Shea, J. , H. Johansen‐Berg , D. Trief , S. Göbel , and M. F. Rushworth . 2007. “Functionally Specific Reorganization in Human Premotor Cortex.” Neuron 54, no. 3: 479–490. 10.1016/j.neuron.2007.04.021. 17481399

[brb371584-bib-0041] Oldfield, R. C. 1971. “The Assessment and Analysis of Handedness: The Edinburgh Inventory.” Neuropsychologia 9, no. 1: 97–113. 10.1016/0028-3932(71)90067-4. 5146491

[brb371584-bib-0042] Putman, P. , B. Verkuil , E. Arias‐Garcia , I. Pantazi , and C. van Schie . 2014. “EEG Theta/Beta Ratio as a Potential Biomarker for Attentional Control and Resilience Against Deleterious Effects of Stress on Attention.” Cognitive, Affective, & Behavioral Neuroscience 14, no. 2: 782–791. 10.3758/s13415-013-0238-7. 24379166

[brb371584-bib-0043] Rossi, S. , M. Hallett , P. M. Rossini , and A. Pascual‐Leone . 2009. “Safety, Ethical Considerations, and Application Guidelines for the Use of Transcranial Magnetic Stimulation in Clinical Practice and Research.” Clinical Neurophysiology 120, no. 12: 2008–2039. 10.1016/j.clinph.2009.08.016. 19833552 PMC3260536

[brb371584-bib-0044] Ruff, C. C. , J. Driver , and S. Bestmann . 2009. “Combining TMS and fMRI: From ‘Virtual Lesions’ to Functional‐Network Accounts of Cognition.” Cortex 45, no. 9: 1043–1049. 10.1016/j.cortex.2008.10.012. 19166996 PMC2726131

[brb371584-bib-0045] Schutte, I. , J. L. Kenemans , and D. J. Schutter . 2017. “Resting‐State Theta/Beta EEG Ratio Is Associated With Reward‐ and Punishment‐Related Reversal Learning.” Cognitive, Affective, & Behavioral Neuroscience 17, no. 4: 754–763. 10.3758/s13415-017-0510-3. PMC5548847 28585018

[brb371584-bib-0046] Schutter, D. , and J. L. Kenemans . 2022. “Theta‐Beta Power Ratio: An Electrophysiological Signature of Motivation, Attention and Cognitive Control.” In The Oxford Handbook of EEG Frequency, 352–376. Oxford Academic. 10.1093/oxfordhb/9780192898340.001.0001.

[brb371584-bib-0047] Schutter, D. J. , and J. Van Honk . 2005. “Electrophysiological Ratio Markers for the Balance Between Reward and Punishment.” Cognitive Brain Research 24, no. 3: 685–690. 10.1016/j.cogbrainres.2005.04.002. 15878265

[brb371584-bib-0048] Siebner, H. R. , T. O. Bergmann , S. Bestmann , et al. 2009. “Consensus Paper: Combining Transcranial Stimulation With Neuroimaging.” Brain Stimulation 2, no. 2: 58–80. 10.1016/j.brs.2008.11.002. 20633405

[brb371584-bib-0049] Siebner, H. R. , G. Hartwigsen , T. Kassuba , and J. C. Rothwell . 2009. “How Does Transcranial Magnetic Stimulation Modify Neuronal Activity in the Brain? Implications for Studies of Cognition.” Cortex 45, no. 9: 1035–1042. 10.1016/j.cortex.2009.02.007. 19371866 PMC2997692

[brb371584-bib-0050] Silvanto, J. , and Z. Cattaneo . 2017. “Common Framework for “Virtual Lesion” and State‐Dependent TMS: The Facilitatory/Suppressive Range Model of Online TMS Effects on Behavior.” Brain and Cognition 119: 32–38. 10.1016/j.bandc.2017.09.007. 28963993 PMC5652969

[brb371584-bib-0051] Silvanto, J. , N. Muggleton , and V. Walsh . 2008. “State‐Dependency in Brain Stimulation Studies of Perception and Cognition.” Trends in Cognitive Sciences 12, no. 12: 447–454. 10.1016/j.tics.2008.09.004. 18951833

[brb371584-bib-0052] Spitzer, B. , and S. Haegens . 2017. “Beyond the Status Quo: A Role for Beta Oscillations in Endogenous Content (Re)activation.” eNeuro 4, no. 4: ENEURO.0170–0117.2017. 10.1523/ENEURO.0170-17.2017. 28785729 PMC5539431

[brb371584-bib-0053] Spronk, D. , M. Arns , A. Bootsma , R. van Ruth , and P. B. Fitzgerald . 2008. “Long Term Effects of Left Frontal rTMS on EEG and ERPs in Patients With Depression.” Clinical EEG and Neuroscience 39, no. 3: 118–124. 10.1177/155005940803900305. 18751560

[brb371584-bib-0054] Studer, B. , A. Pedroni , and J. Rieskamp . 2013. “Predicting Risk‐Taking Behavior From Prefrontal Resting‐State Activity and Personality.” PLoS ONE 8, no. 10: e76861. 10.1371/journal.pone.0076861. 24116176 PMC3792091

[brb371584-bib-0055] Thut, G. , and A. Pascual‐Leone . 2010. “A Review of Combined TMS‐EEG Studies to Characterize Lasting Effects of Repetitive TMS and Assess Their Usefulness in Cognitive and Clinical Neuroscience.” Brain topography 22, no. 4: 219–232. 10.1007/s10548-009-0115-4. 19862614 PMC3260526

[brb371584-bib-0056] Thut, G. , P. G. Schyns , and J. Gross . 2011. “Entrainment of Perceptually Relevant Brain Oscillations by Non‐Invasive Rhythmic Stimulation of the Human Brain.” Frontiers in Psychology 2: 170. 10.3389/fpsyg.2011.00170. 21811485 PMC3142861

[brb371584-bib-0057] Tomarken, A. J. , R. J. Davidson , R. E. Wheeler , and R. C. Doss . 1992. “Individual Differences in Anterior Brain Asymmetry and Fundamental Dimensions of Emotion.” Journal of Personality and Social Psychology 62, no. 4: 676–687. 10.1037/0022-3514.62.4.676. 1583591

[brb371584-bib-0058] Tulviste, J. , and T. Bachmann . 2019. “Diminished Risk‐Aversion After Right DLPFC Stimulation: Effects of rTMS on a Risky Ball Throwing Task.” Journal of the International Neuropsychological Society 25, no. 1: 72–78. 10.1017/S1355617718000930. 30520706

[brb371584-bib-0059] Uehara, K. , J. M. Fine , and M. Santello . 2023. “Modulation of Cortical Beta Oscillations Influences Motor Vigor: A Rhythmic TMS‐EEG Study.” Human Brain Mapping 44, no. 3: 1158–1172. 10.1002/hbm.26149. 36419365 PMC9875933

[brb371584-bib-0060] Van't Wout, M. , R. S. Kahn , A. G. Sanfey , and A. Aleman . 2005. “Repetitive Transcranial Magnetic Stimulation Over the Right Dorsolateral Prefrontal Cortex Affects Strategic Decision‐Making.” Neuroreport 16, no. 16: 1849–1852. 16237340 10.1097/01.wnr.0000183907.08149.14

[brb371584-bib-0061] Veniero, D. , D. Brignani , G. Thut , and C. Miniussi . 2011. “Alpha‐Generation as Basic Response‐Signature to Transcranial Magnetic Stimulation (TMS) Targeting the human Resting Motor Cortex: A TMS/EEG Co‐Registration Study.” Psychophysiology 48, no. 10: 1381–1389. 10.1111/j.1469-8986.2011.01218.x. 21542853

[brb371584-bib-0062] Wang, Q. , A. Gong , Z. Feng , Y. Bai , and U. Ziemann . 2024. “Interactions of Transcranial Magnetic Stimulation With Brain Oscillations: A Narrative Review.” Frontiers in systems neuroscience 18: 1489949. 10.3389/fnsys.2024.1489949. 39698203 PMC11652484

[brb371584-bib-0063] Wischnewski, M. , M. L. Joergensen , B. Compen , and D. J. Schutter . 2020. “Frontal Beta Transcranial Alternating Current Stimulation Improves Reversal Learning.” Cerebral Cortex 30, no. 5: 3286–3295. 10.1093/cercor/bhz309. 31898728 PMC7197207

[brb371584-bib-0064] Wong, S. W. , R. H. Chan , and J. N. Mak . 2014. “Spectral Modulation of Frontal EEG During Motor Skill Acquisition: A Mobile EEG Study.” International Journal of Psychophysiology 91, no. 1: 16–21. 10.1016/j.ijpsycho.2013.09.004. 24095979

[brb371584-bib-0065] Xiong, G. , X. Li , Z. Dong , S. Cai , J. Huang , and Q. Li . 2019. “Modulating Activity in the Prefrontal Cortex Changes Intertemporal Choice for Loss: A Transcranial Direct Current Stimulation Study.” Frontiers in Human Neuroscience 13: 167. 10.3389/fnhum.2019.00167. 31178709 PMC6543463

[brb371584-bib-0066] Yaple, Z. , M. Martinez‐Saito , B. Awasthi , M. Feurra , A. Shestakova , and V. Klucharev . 2017. “Transcranial Alternating Current Stimulation Modulates Risky Decision Making in a Frequency‐Controlled Experiment.” eNeuro 4, no. 6: ENEURO.0136–0117.2017. 10.1523/ENEURO.0136-17.2017. 29379865 PMC5779115

[brb371584-bib-0067] Zanto, T. P. , J. Z. Chadick , G. Satris , and A. Gazzaley . 2013. “Rapid Functional Reorganization in Human Cortex Following Neural Perturbation.” Journal of Neuroscience 33, no. 41: 16268–16274. 10.1523/JNEUROSCI.0308-13.2013. 24107958 PMC3792463

